# Publisher Correction: Bidirectional and parallel relationships in macaque face circuit revealed by fMRI and causal pharmacological inactivation

**DOI:** 10.1038/s41467-022-35057-z

**Published:** 2022-11-24

**Authors:** Ning Liu, Marlene Behrmann, Janita N. Turchi, Galia Avidan, Fadila Hadj-Bouziane, Leslie G. Ungerleider

**Affiliations:** 1 Section on Neurocircuitry, Laboratory of Brain and Cognition, NIMH, NIH, Bethesda, MD 20892 USA; 2 State Key Laboratory of Brain and Cognitive Science, Institute of Biophysics, Chinese Academy of Sciences, 100101 Beijing, China; 3 Department of Ophthalmology, University of Pittsburgh, Pittsburgh, PA 15213 USA; 4 Department of Psychology and Neuroscience Institute, Carnegie Mellon University, Pittsburgh, PA 15213 USA; 5 Laboratory of Neuropsychology, NIMH, NIH, Bethesda, MD 20892 USA; 6 Department of Psychology, Ben-Gurion University of the Negev, Beer-Sheva, 8410501 Israel; 7 INSERM, U1028, CNRS UMR5292, Lyon Neuroscience Research Center, ImpAct Team, F-69000 Lyon, France; 8 University UCBL Lyon 1, F-69000 Lyon, France

**Keywords:** Object vision, Neural circuits

Correction to: *Nature Communications* 10.1038/s41467-022-34451-x, published online 09 November 2022

The wrong Supplementary file was originally published with this article; it has now been replaced with the correct. The original article has been corrected.

## Supplementary information

Updated Supplementary Information

