# Supplementary material for: Bidirectional and parallel relationships in macaque face circuit revealed by fMRI and causal pharmacological inactivation
Source: Nat Commun. 2022 Nov 9;13:6787. doi: 10.1038/s41467-022-34451-x (PMC9646786; doi:10.1038/s41467-022-34451-x)
Supplement: Supplementary file 1 — Supplementary Information [file 41467_2022_34451_MOESM1_ESM.pdf]

Bidirectional and parallel relationships in macaque face circuit revealed by fMRI and causal pharmacological inactivation

Supplementary Tables

Table S1 GLMM results of main effects of Treatment and Hemisphere on each of the four IT face patches and the amygdala following inactivation of the middle face patches. The name of the ROI is shown in the leftmost column.

		Responses to Faces						Responses to Objects					
	df2	Treatment		Hemisphere		Interaction		Treatment		Hemisphere		Interaction	
		F	p	*F*	p	*F*	p	*F*	p	*F*	p	*F*	p
MF	656	37.17	<0.001	214.90	<0.001	42.43	<0.001	12.60	<0.001	7.33	0.007	6.19	<0.001
ML	656	32.81	<0.001	312.95	<0.001	66.95	<0.001	8.04	<0.001	20.39	<0.001	13.75	<0.001
AF	656	8.68	<0.001	97.45	<0.001	20.34	<0.001	1.86	0.134	24.77	<0.001	10.21	<0.001
AL	558	5.17	0.002	17.50	<0.001	5.31	0.001	2.03	0.108	1.18	0.278	1.28	0.281
AMG	460	1.06	0.365	8.95	0.003	4.15	0.006	3.77	0.011	8.44	0.004	2.58	0.053

Table S2 Volumetric overlap between the middle face patches and their inactivation injections.

	MF			ML		
	Injection Overlap (%) Mean±SD Range	ROI Overlap (%) Mean±SD Range	Theoretical ROI Overlap (%)	Injection Overlap (%) Mean±SD Range	ROI Overlap (%) Mean±SD Range	Theoretical ROI Overlap (%)
**Monkey C**						
**Left Hemisphere**						
MF&ML Inactivation	NA	NA	NA	NA	NA	NA
MF Inactivation	NA	NA	NA	NA	NA	NA
ML Inactivation	0	0	0	96.79±1.48<br>95.00~98.86	38.49±2.55<br>34.82~41.35	39.75
**Right Hemisphere**						
MF&ML Inactivation	80.57±10.35<br>73.25~87.89	44.71±5.29<br>40.97~48.45	55.52	90.16±12.22<br>81.52~98.80	52.00±5.96<br>47.78~56.21	57.75
MF Inactivation	92.31	48.33	52.36	0	0	0
ML Inactivation	0	0	0	87.65±2.28<br>86.03~89.26	50.29±2.58<br>48.46~52.11	57.35
**Monkey D**						
**Left Hemisphere**						
MF&ML Inactivation	91.95±3.61<br>89.40~94.50	64.58±1.77<br>63.33~65.83	70.25	71.97±3.88<br>69.22~74.71	42.08±1.69<br>40.88~43.27	58.62
MF Inactivation	90.90±2.48<br>88.98~94.43	62.92±1.60<br>61.56~64.95	69.22	6.90±2.68<br>2.12~8.35	4.08±1.58<br>1.26~4.91	0
ML Inactivation	20.61	13.99	0	72.02	41.76	57.99
**Right Hemisphere**						
MF&ML Inactivation	NA	NA	NA	NA	NA	NA
MF Inactivation	NA	NA	NA	NA	NA	NA
ML Inactivation	0.36±0.63<br>0~1.09	0.26±0.44<br>0~0.77	0	96.61±3.71<br>92.36~99.14	49.65±2.87<br>46.43~51.92	51.37
**Across All the Hemispheres**	88.93±5.61<br>80.57~92.31	55.14±10.08<br>44.71~64.58	61.84±9.22<br>52.36~70.25	85.87±11.32<br>71.97~96.79	45.71±5.60<br>38.49~52.00	53.81±7.38<br>39.75~58.62

Table S3 Volumetric overlap between the anterior face patches and their inactivation injections.

	AF			AL		
	Injection Overlap (%) Mean±SD Range	ROI Overlap (%) Mean±SD Range	Theoretical ROI Overlap (%)	Injection Overlap (%) Mean±SD Range	ROI Overlap (%) Mean±SD Range	Theoretical ROI Overlap (%)
**Monkey C**						
**Left Hemisphere**						
AF&AL Inactivation	82.97±0.20<br>82.83~83.11	41.57±7.71<br>36.12~47.02	50.11	85.58±1.84<br>84.28~86.88	38.74±5.76<br>34.67~42.81	45.21
AF Inactivation	72.02±11.47<br>58.79~79.10	34.02±7.22<br>25.69~38.19	46.94	0	0	0
AL Inactivation	NA	NA	NA	NA	NA	NA
**Right Hemisphere**						
AF&AL Inactivation	88.39±0.79<br>87.70~89.49	39.60±4.91<br>34.65~45.76	44.83	76.64±12.10<br>69.53~94.74	34.06±1.99<br>32.00~36.67	45.33
AF Inactivation	NA	NA	NA	NA	NA	NA
AL Inactivation	NA	NA	NA	NA	NA	NA
**Monkey D**						
**Left Hemisphere**						
AF&AL Inactivation	73.54±3.61<br>70.98~76.09	51.97±0.79<br>51.41~52.53	70.73	77.19±0.95<br>76.52~77.86	42.13±4.24<br>39.13~45.13	54.55
AF Inactivation	79.31±4.91<br>75.84~82.78	41.56±12.87<br>32.46~50.66	53.00	0	0	0
AL Inactivation	0	0	0	70.50±7.88<br>64.93~76.07	50.62±19.47<br>36.85~64.39	73.81
**Right Hemisphere**						
AF&AL Inactivation	NA	NA	NA	NA	NA	NA
AF Inactivation	NA	NA	NA	NA	NA	NA
AL Inactivation	1.46	1.02	0	57.80	38.66	66.90
**Across All the Hemispheres**	79.25±6.75<br>72.02~88.39	41.74±6.50<br>34.02~51.97	53.12±10.32<br>44.83~70.73	73.54±10.31<br>57.80~85.58	40.84±6.17<br>34.06~50.62	57.16±12.86<br>45.21~73.81

Table S4 GLMM results of main effects of Treatment and Hemisphere on each of the four IT face patches and the amygdala following inactivation of the anterior face patches. The name of the ROI is shown in the leftmost column.

		Responses to Faces						Responses to Objects					
	df2	Treatment		Hemisphere		Interaction		Treatment		Hemisphere		Interaction	
		F	p	*F*	p	*F*	p	*F*	p	*F*	p	*F*	p
MF	478	6.02	<0.001	33.90	<0.001	11.02	<0.001	6.89	<0.001	0.57	0.450	1.42	0.237
ML	478	2.01	0.111	104.73	<0.001	22.87	<0.001	4.49	0.004	7.14	0.008	5.09	0.002
AF	478	35.50	<0.001	70.78	<0.001	16.41	<0.001	2.83	0.038	3.05	0.082	1.63	0.181
AL	328	14.11	<0.001	63.51	<0.001	19.51	<0.001	2.93	0.034	37.66	<0.001	4.01	0.008
AMG	328	5.37	0.001	30.26	<0.001	14.51	<0.001	5.83	0.001	5.13	0.024	21.66	<0.001

Table S5 Summary of the Different Types of Face Patch Inactivations. Data are presented as the total number of runs (and the total number of sessions) for analyses on responses to faces/objects.

	Monkey C LHem	Monkey C RHem	Monkey D LHem	Monkey D RHem	Total
M-Control	50(6)	26(3)	31(4)	50(6)	157(19)
MF&ML Inactivation		16(2)	16(2)		32(4)
MF Inactivation		10(1)	43(5)		53(6)
ML Inactivation	42(5)	14(2)	8(1)	26(3)	90(11)
A-Control	28(3)	22(3)	24(3)	32(4)	106(13)
AF&AL Inactivation	17(2)	34(4)	17(2)		68(8)
AF Inactivation	27(3)		16(2)		43(5)
AL Inactivation			18(2)	8(1)	26(3)

Supplementary Figures

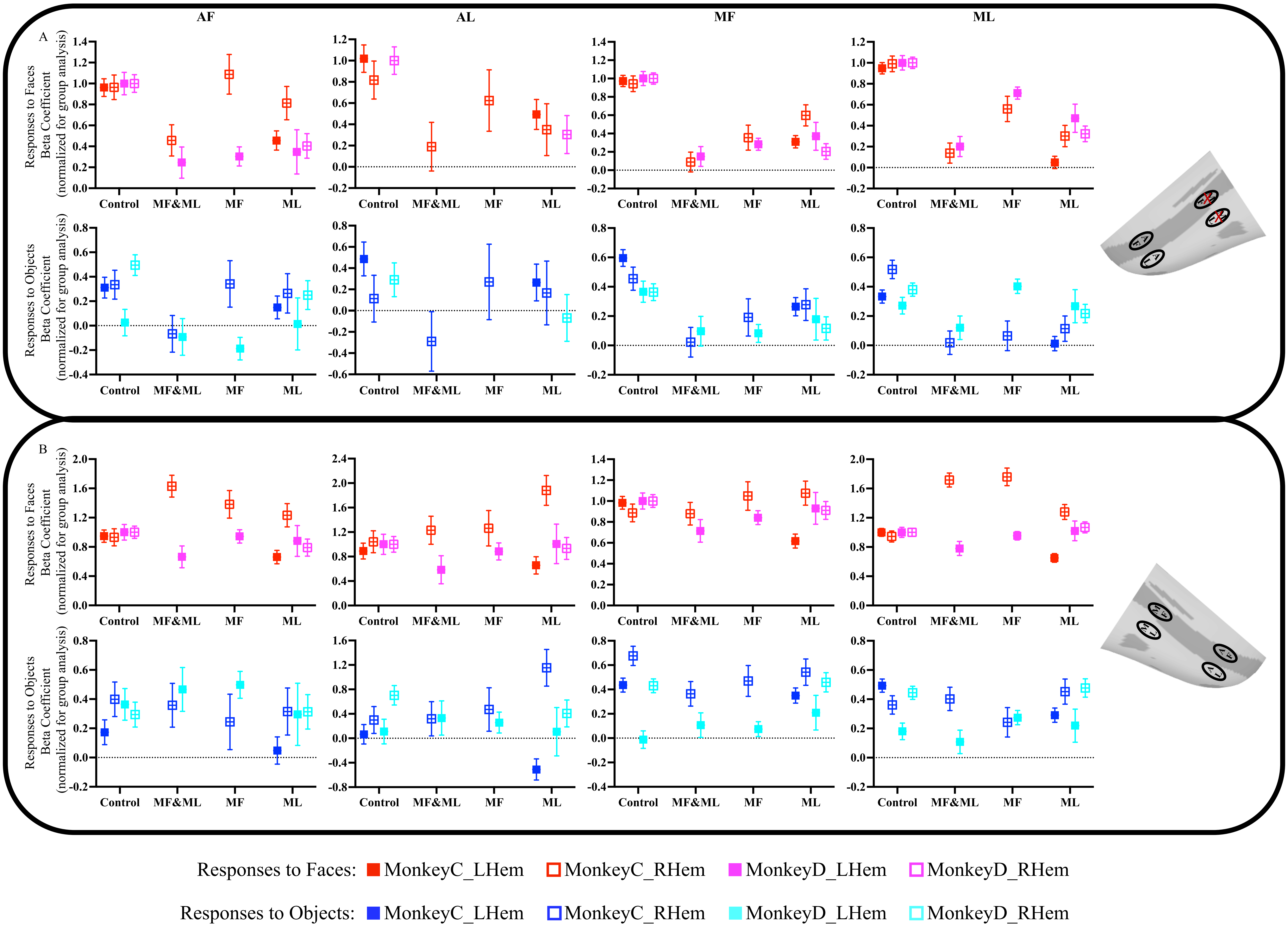

Figure S1. Effects of middle face patches inactivation on responses to faces (top rows in each panel) and objects (bottom rows in each panel) in the ipsilateral (A) and contralateral (B) hemispheres in Monkeys C and D. The schematic diagrams to A&B left indicate the corresponding hemisphere. The name of the ROI is shown at the top of each panel. MF&ML: combined MF and ML inactivation; MF: MF alone inactivation; ML: ML alone inactivation. Box plots display mean values +/- SEM. The location of the inactivation is marked with red X. AF, anterior fundal patch; AL, anterior lateral patch; MF, middle fundal patch; ML, middle lateral patch.

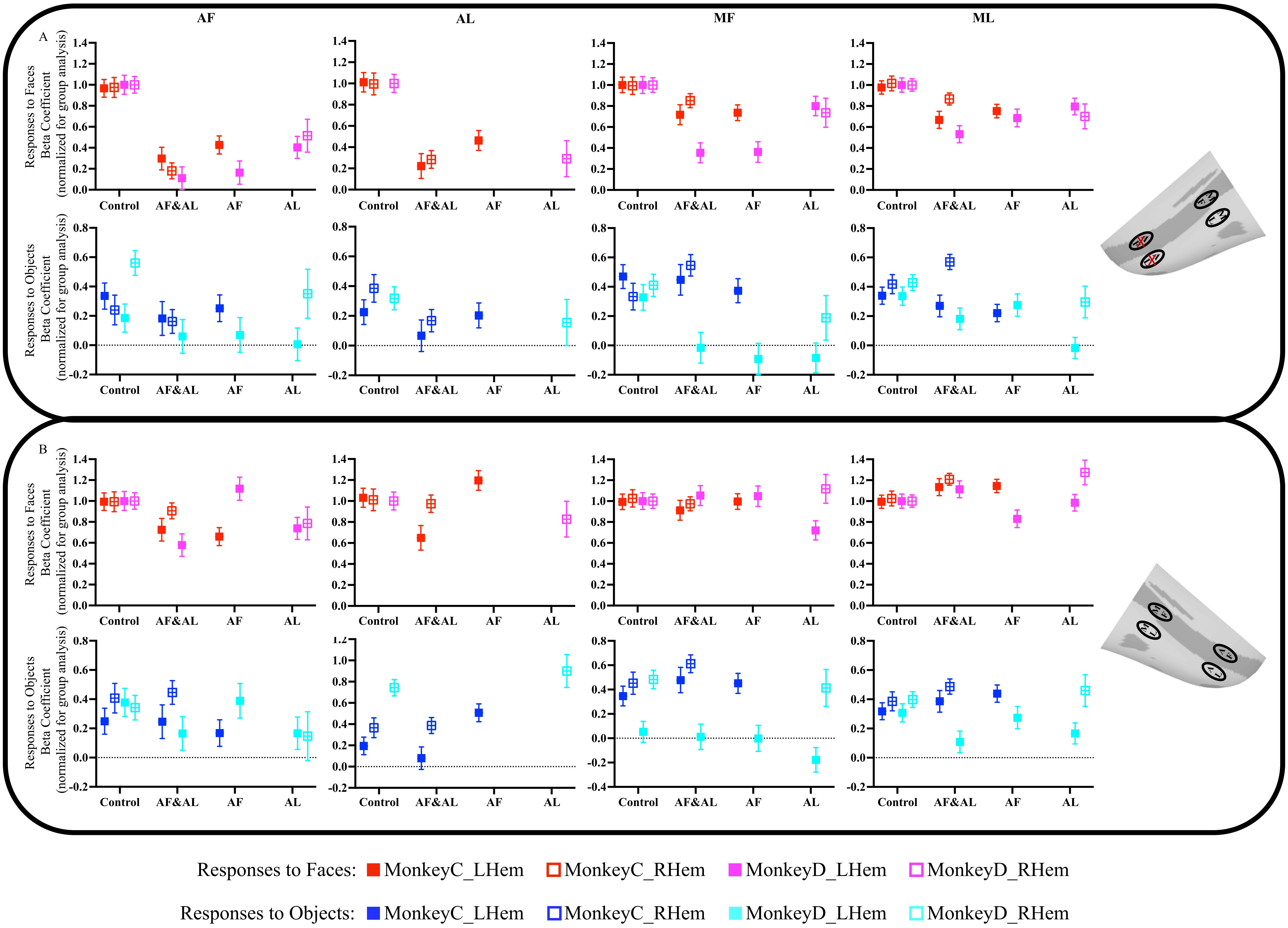

Figure S2. Effects of anterior face patches inactivation on responses to faces (top rows in each panel) and objects (bottom rows in each panel) in the ipsilateral (A) and contralateral (B) hemispheres in Monkeys C and D. The schematic diagrams to A&B left indicate the corresponding hemisphere. The name of the ROI is shown at the top of each panel. AF&AL: combined AF and AL inactivation; AF: AF alone inactivation; AL: AL alone inactivation. Box plots display mean values +/- SEM. The location of the inactivation is marked with red X. AF, anterior fundal patch; AL, anterior lateral patch; MF, middle fundal patch; ML, middle lateral patch.

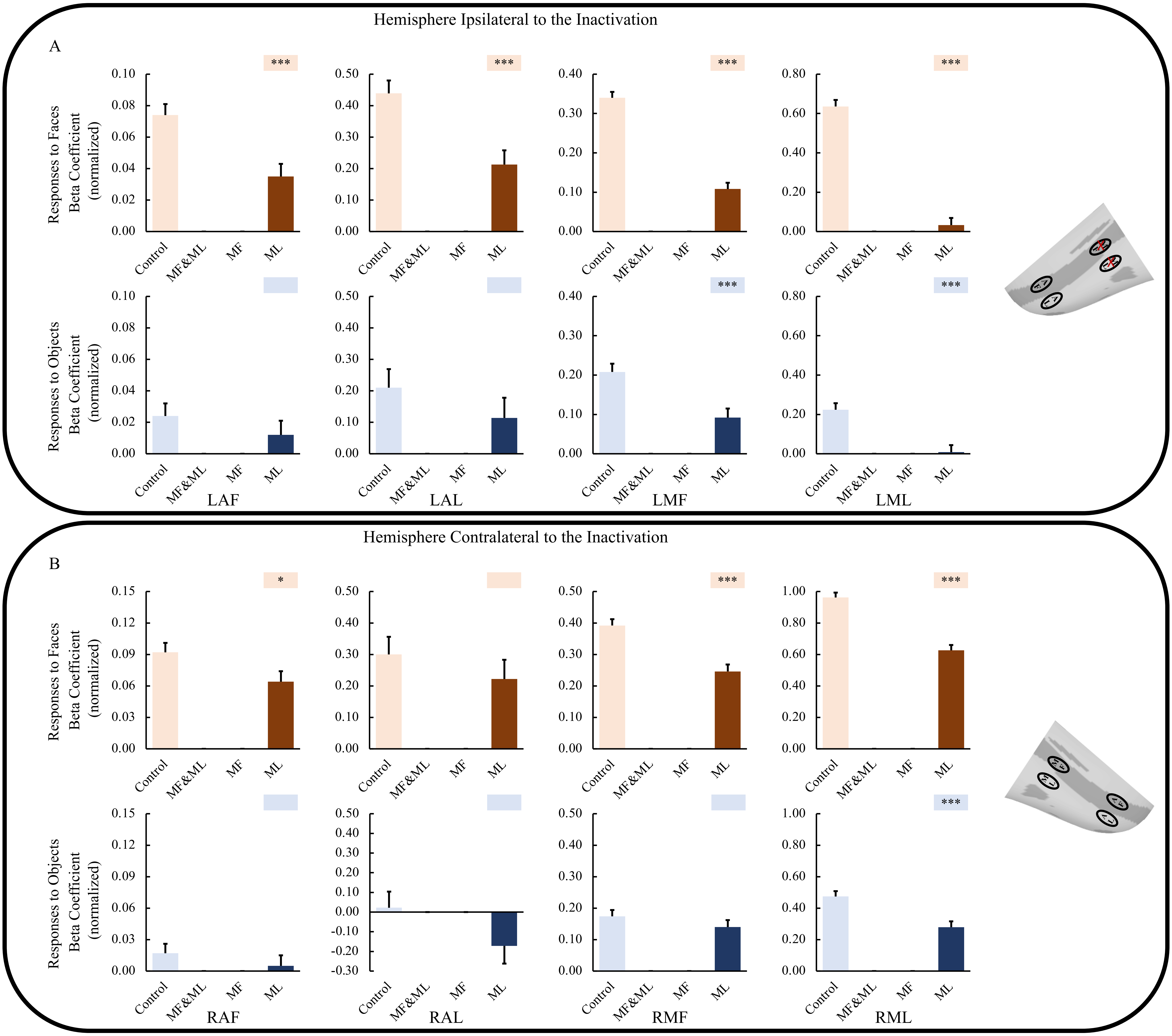

Figure S3. Effects of left middle face patches inactivation on responses to faces (top rows in each panel) and objects (bottom rows in each panel) in the ipsilateral (A) and contralateral (B) hemispheres in Monkey C. The name of the ROI is shown at the bottom of each panel. MF&ML: combined MF and ML inactivation; MF: MF alone inactivation; ML: ML alone inactivation. Generalized Linear Mixed Models were performed with Treatment (Control: n=50, ML: n=42) as fixed factors, and Session and Run as random factors. Post hoc testing was done with correction for multiple testing using the Holm-Bonferroni method. Color bars above a particular histogram contain the statistical results of comparing this inactivation condition and the condition to its left with the same color as the bar: *, significant difference from control (* $p<0.05$, *** $p<0.001$, in light red/blue boxes); value, uncorrected p value; empty bar, no significant difference no matter whether multiple comparison corrections were done or not. Box plots display mean values +/- SEM. The location of the inactivation is marked with red X. AF, anterior fundal patch; AL, anterior lateral patch; MF, middle fundal patch; ML, middle lateral patch.

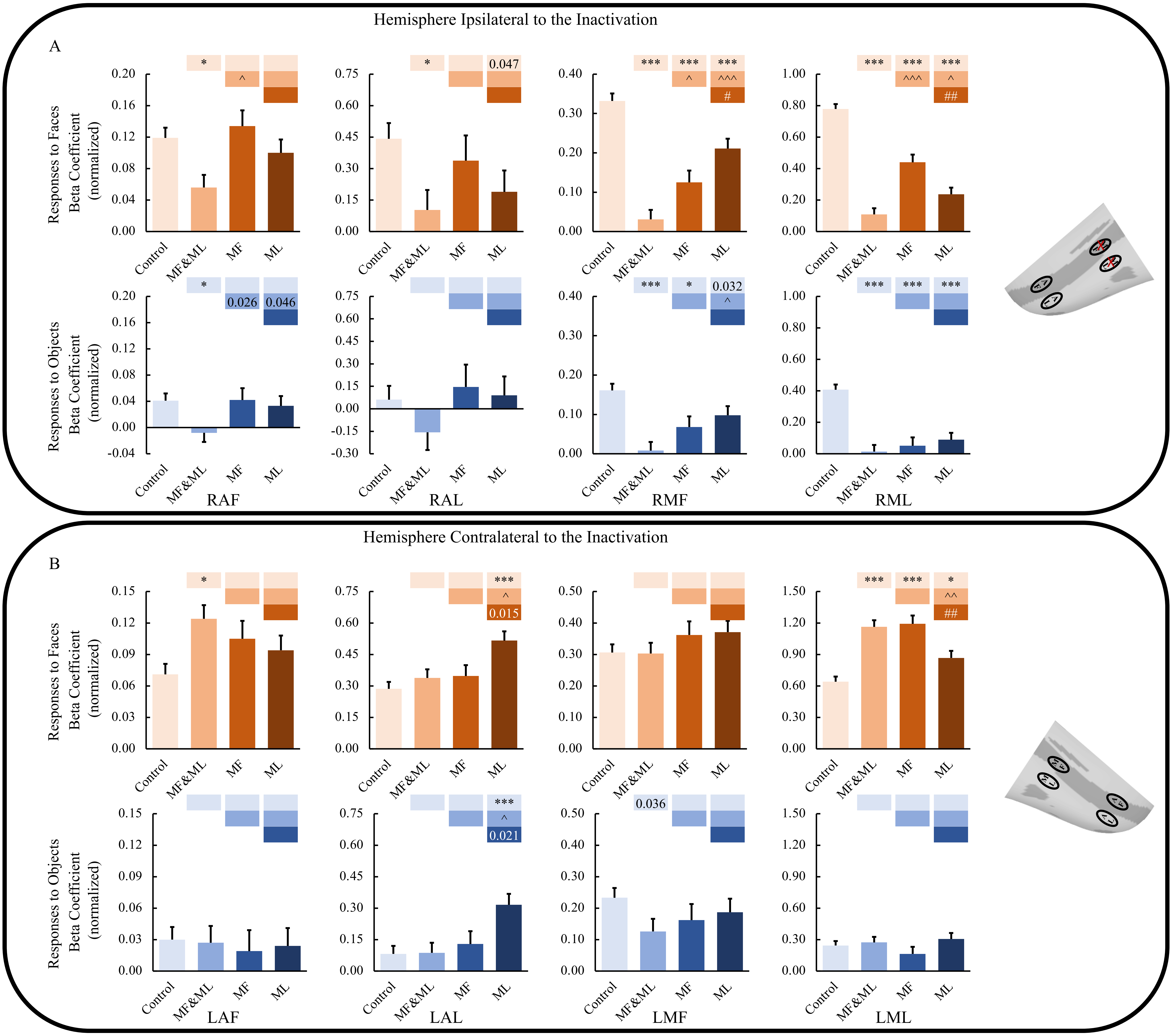

Figure S4. Effects of right middle face patches inactivation on responses to faces (top rows in each panel) and objects (bottom rows in each panel) in the ipsilateral (A) and contralateral (B) hemispheres in Monkey C. The name of the ROI is shown at the bottom of each panel. MF&ML: combined MF and ML inactivation; MF: MF alone inactivation; ML: ML alone inactivation. Generalized Linear Mixed Models were performed with Treatment (Control: n=26, MF&ML: n=16, MF: n=10, ML: n=14) as fixed factors, and Session and Run as random factors. Post hoc testing was done with correction for multiple testing using the Holm-Bonferroni method. Color bars above a particular histogram contain the statistical results of comparing this inactivation condition and the condition to its left with the same color as the bar: *, significant difference from control (* p<0.05, *** p<0.001, in light red/blue boxes); ^, significant difference from combined MF and ML inactivation (^ p<0.05, ^^ p<0.01, ^^^ p<0.001, in middle red/blue boxes); #, significant difference from MF alone inactivation (# p<0.05, ## p<0.01, in dark red/blue boxes); value, uncorrected p value; empty bar, no significant difference no matter whether multiple comparison corrections were done or not. Box plots display mean values +/- SEM. The location of the inactivation is marked with red X. AF, anterior fundal patch; AL, anterior lateral patch; MF, middle fundal patch; ML, middle lateral patch.

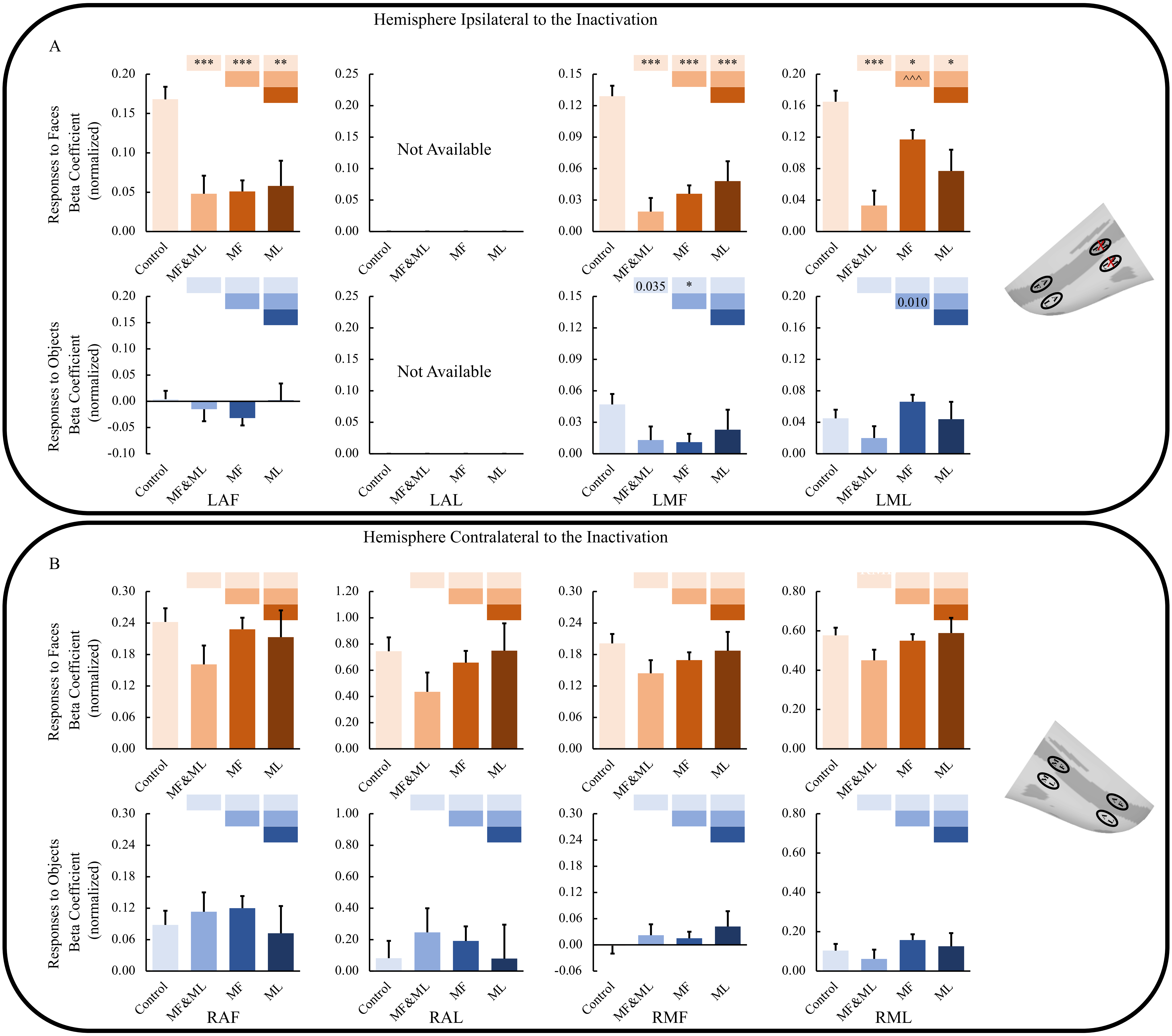

Figure S5. Effects of left middle face patches inactivation on responses to faces (top rows in each panel) and objects (bottom rows in each panel) in the ipsilateral (A) and contralateral (B) hemispheres in Monkey D. The name of the ROI is shown at the bottom of each panel. MF&ML: combined MF and ML inactivation; MF: MF alone inactivation; ML: ML alone inactivation. Generalized Linear Mixed Models were performed with Treatment (Control: n=31, MF&ML: n=16, MF: n=43, ML: n=8) as fixed factors, and Session and Run as random factors. Post hoc testing was done with correction for multiple testing using the Holm-Bonferroni method. Color bars above a particular histogram contain the statistical results of comparing this inactivation condition and the condition to its left with the same color as the bar: *, significant difference from control (* p<0.05, ** p<0.01, *** p<0.001, in light red/blue boxes); ^, significant difference from combined MF and ML inactivation (^^^ p<0.001, in middle red/blue boxes); value, uncorrected p value; empty bar, no significant difference no matter whether multiple comparison corrections were done or not. Box plots display mean values +/- SEM. The location of the inactivation is marked with red X. AF, anterior fundal patch; AL, anterior lateral patch; MF, middle fundal patch; ML, middle lateral patch.

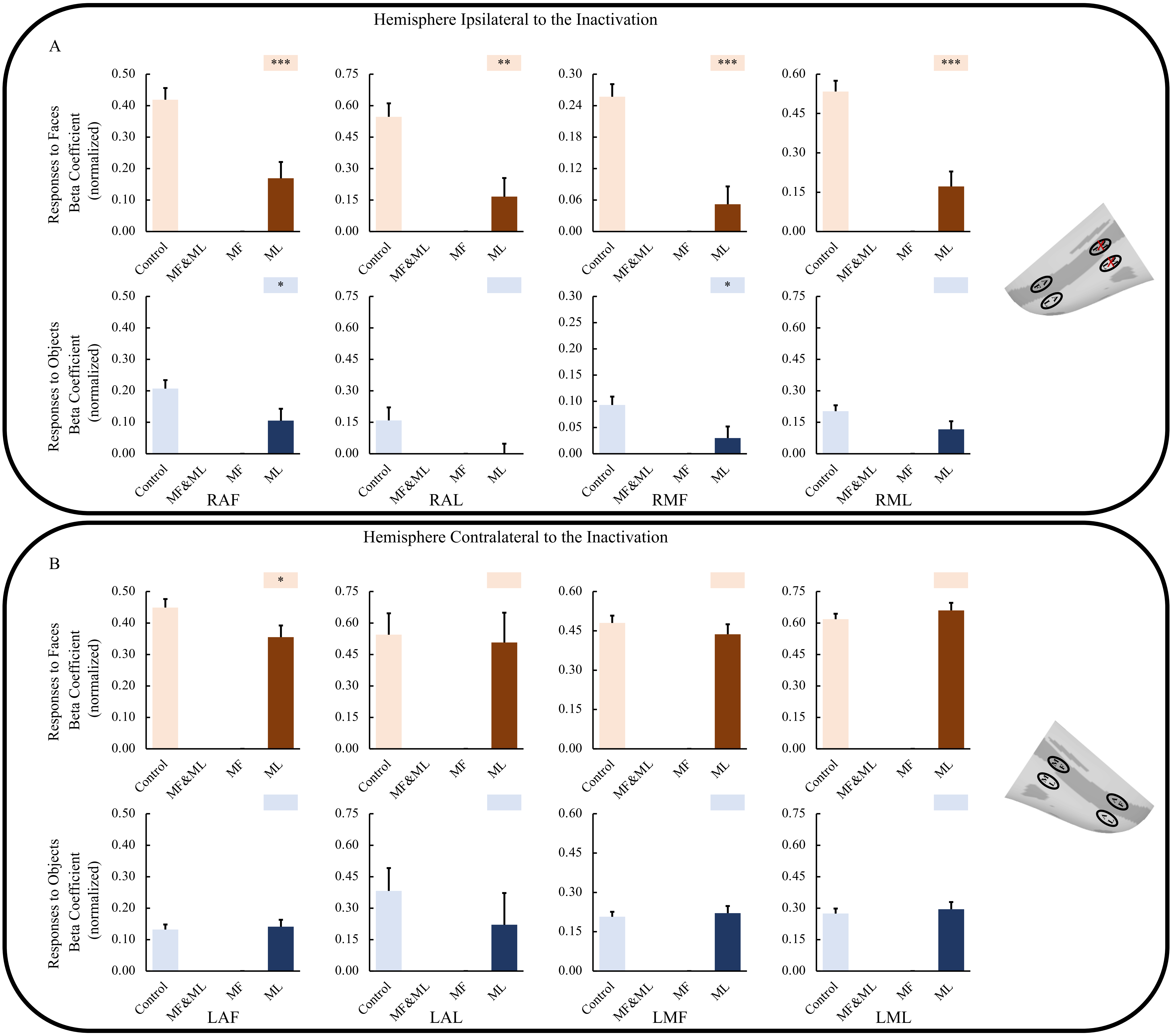

Figure S6. Effects of right middle face patches inactivation on responses to faces (top rows in each panel) and objects (bottom rows in each panel) in the ipsilateral (A) and contralateral (B) hemispheres in Monkey D. The name of the ROI is shown at the bottom of each panel. MF&ML: combined MF and ML inactivation; MF: MF alone inactivation; ML: ML alone inactivation. Generalized Linear Mixed Models were performed with Treatment (Control: n=50, ML: n=26) as fixed factors, and Session and Run as random factors. Post hoc testing was done with correction for multiple testing using the Holm-Bonferroni method. Color bars above a particular histogram contain the statistical results of comparing this inactivation condition and the condition to its left with the same color as the bar: *, significant difference from control (* $p<0.05$, ** $p<0.01$, *** $p<0.001$, in light red/blue boxes); value, uncorrected p value; empty bar, no significant difference no matter whether multiple comparison corrections were done or not. Box plots display mean values +/- SEM. The location of the inactivation is marked with red X. AF, anterior fundal patch; AL, anterior lateral patch; MF, middle fundal patch; ML, middle lateral patch.

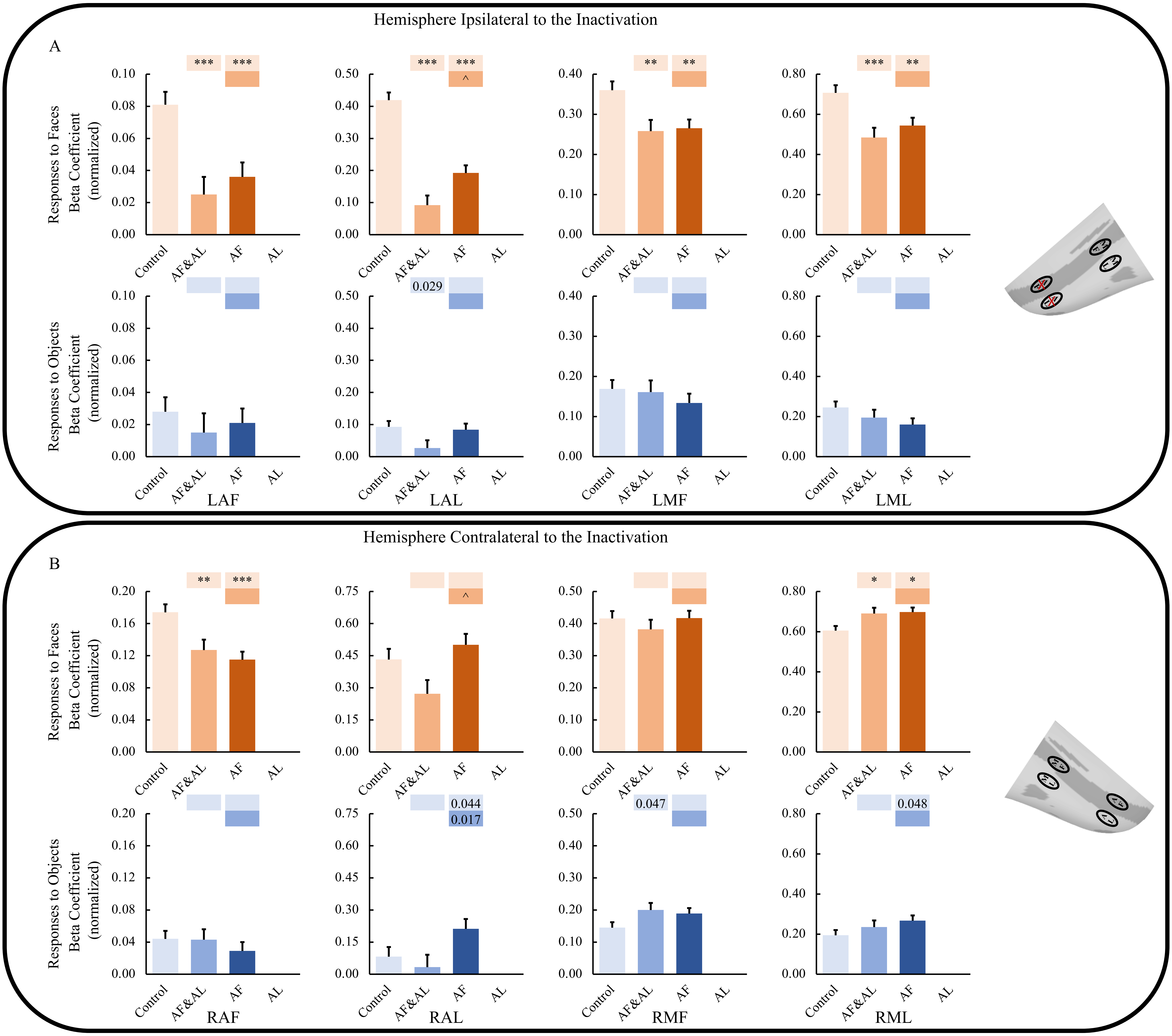

Figure S7. Effects of left anterior face patches inactivation on responses to faces (top rows in each panel) and objects (bottom rows in each panel) in the ipsilateral (A) and contralateral (B) hemispheres in Monkey C. The name of the ROI is shown at the bottom of each panel. AF&AL: combined AF and AL inactivation; AF: AF alone inactivation; AL: AL alone inactivation. Generalized Linear Mixed Models were performed with Treatment (Control: n=28, AF&AL: n=17, AF: n =27) as fixed factors, and Session and Run as random factors. Post hoc testing was done with correction for multiple testing using the Holm-Bonferroni method. Color bars above a particular histogram contain the statistical results of comparing this inactivation condition and the condition to its left with the same color as the bar: *, significant difference from control (* $p<0.05$, ** $p<0.01$, *** $p<0.001$, in light red/blue boxes); ^, significant difference from combined AF and AL inactivation (^ $p<0.05$, in middle red/blue boxes); value, uncorrected p value; empty bar, no significant difference no matter whether multiple comparison corrections were done or not. Box plots display mean values +/- SEM. The location of the inactivation is marked with red X. AF, anterior fundal patch; AL, anterior lateral patch; MF, middle fundal patch; ML, middle lateral patch.

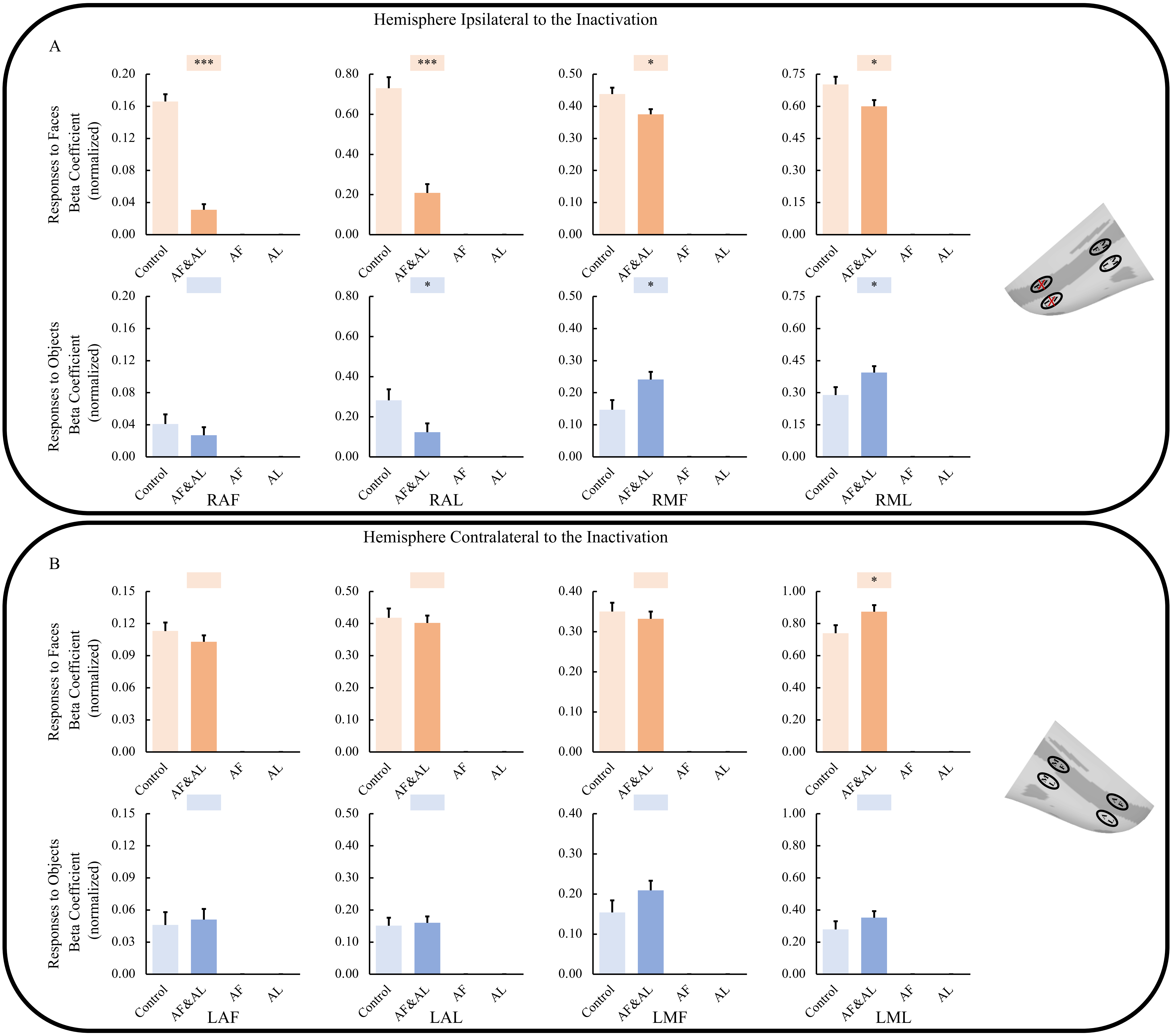

Figure S8. Effects of right anterior face patches inactivation on responses to faces (top rows in each panel) and objects (bottom rows in each panel) in the ipsilateral (A) and contralateral (B) hemispheres in Monkey C. The name of the ROI is shown at the bottom of each panel. AF&AL: combined AF and AL inactivation; AF: AF alone inactivation; AL: AL alone inactivation. Generalized Linear Mixed Models were performed with Treatment (Control: n=22, AF&AL: n=34) as fixed factors, and Session and Run as random factors. Post hoc testing was done with correction for multiple testing using the Holm-Bonferroni method. Color bars above a particular histogram contain the statistical results of comparing this inactivation condition and the condition to its left with the same color as the bar: *, significant difference from control (* $p<0.05$, *** $p<0.001$, in light red/blue boxes); value, uncorrected p value; empty bar, no significant difference no matter whether multiple comparison corrections were done or not. Box plots display mean values +/- SEM. The location of the inactivation is marked with red X. AF, anterior fundal patch; AL, anterior lateral patch; MF, middle fundal patch; ML, middle lateral patch.

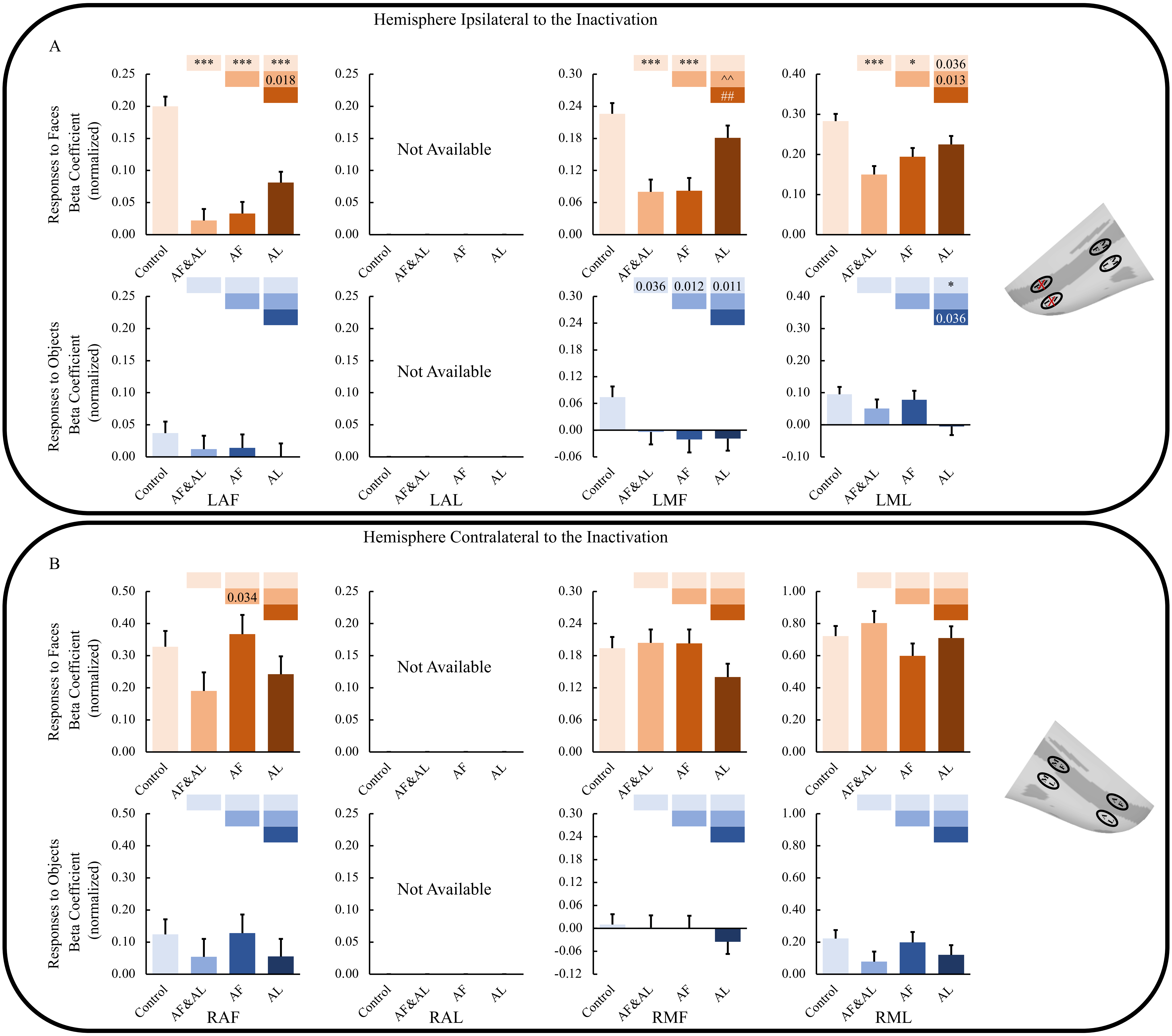

Figure S9. Effects of left anterior face patches inactivation on responses to faces (top rows in each panel) and objects (bottom rows in each panel) in the ipsilateral (A) and contralateral (B) hemispheres in Monkey D. The name of the ROI is shown at the bottom of each panel. AF&AL: combined AF and AL inactivation; AF: AF alone inactivation; AL: AL alone inactivation. Generalized Linear Mixed Models were performed with Treatment (Control: n=24, AF&AL: n=17, AF: n=16, AL: n=18) as fixed factors, and Session and Run as random factors. Post hoc testing was done with correction for multiple testing using the Holm-Bonferroni method. Color bars above a particular histogram contain the statistical results of comparing this inactivation condition and the condition to its left with the same color as the bar: *, significant difference from control (* $p<0.05$, *** $p<0.001$, in light red/blue boxes); ^, significant difference from combined AF and AL inactivation (^^ $p<0.01$, in middle red/blue boxes); #, significant difference from AF alone inactivation (## $p<0.01$, in dark red/blue boxes); value, uncorrected p value; empty bar, no significant difference no matter whether multiple comparison corrections were done or not. Box plots display mean values +/- SEM. The location of the inactivation is marked with red X. AF, anterior fundal patch; AL, anterior lateral patch; MF, middle fundal patch; ML, middle lateral patch.

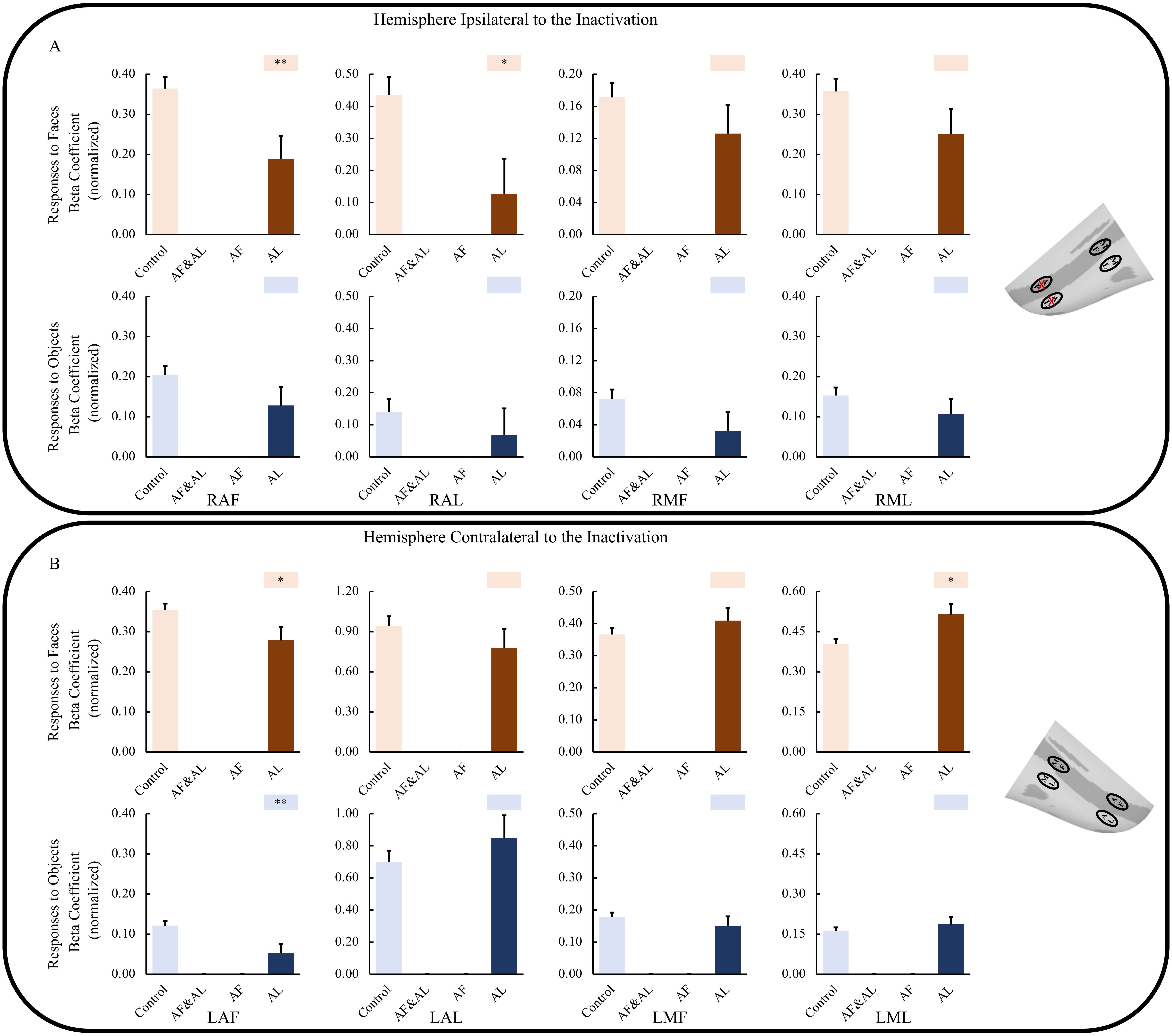

Figure S10. Effects of right anterior face patches inactivation on responses to faces (top rows in each panel) and objects (bottom rows in each panel) in the ipsilateral (A) and contralateral (B) hemispheres in Monkey D. The name of the ROI is shown at the bottom of each panel. AF&AL: combined AF and AL inactivation; AF: AF alone inactivation; AL: AL alone inactivation. Generalized Linear Mixed Models were performed with Treatment (Control: n=32, AL: n=8) as fixed factors, and Session and Run as random factors. Post hoc testing was done with correction for multiple testing using the Holm-Bonferroni method. Color bars above a particular histogram contain the statistical results of comparing this inactivation condition and the condition to its left with the same color as the bar: *, significant difference from control (* $p<0.05$, ** $p<0.01$, in light red/blue boxes); empty bar, no significant difference no matter whether multiple comparison corrections were done or not. Box plots display mean values +/- SEM. The location of the inactivation is marked with red X. AF, anterior fundal patch; AL, anterior lateral patch; MF, middle fundal patch; ML, middle lateral patch.

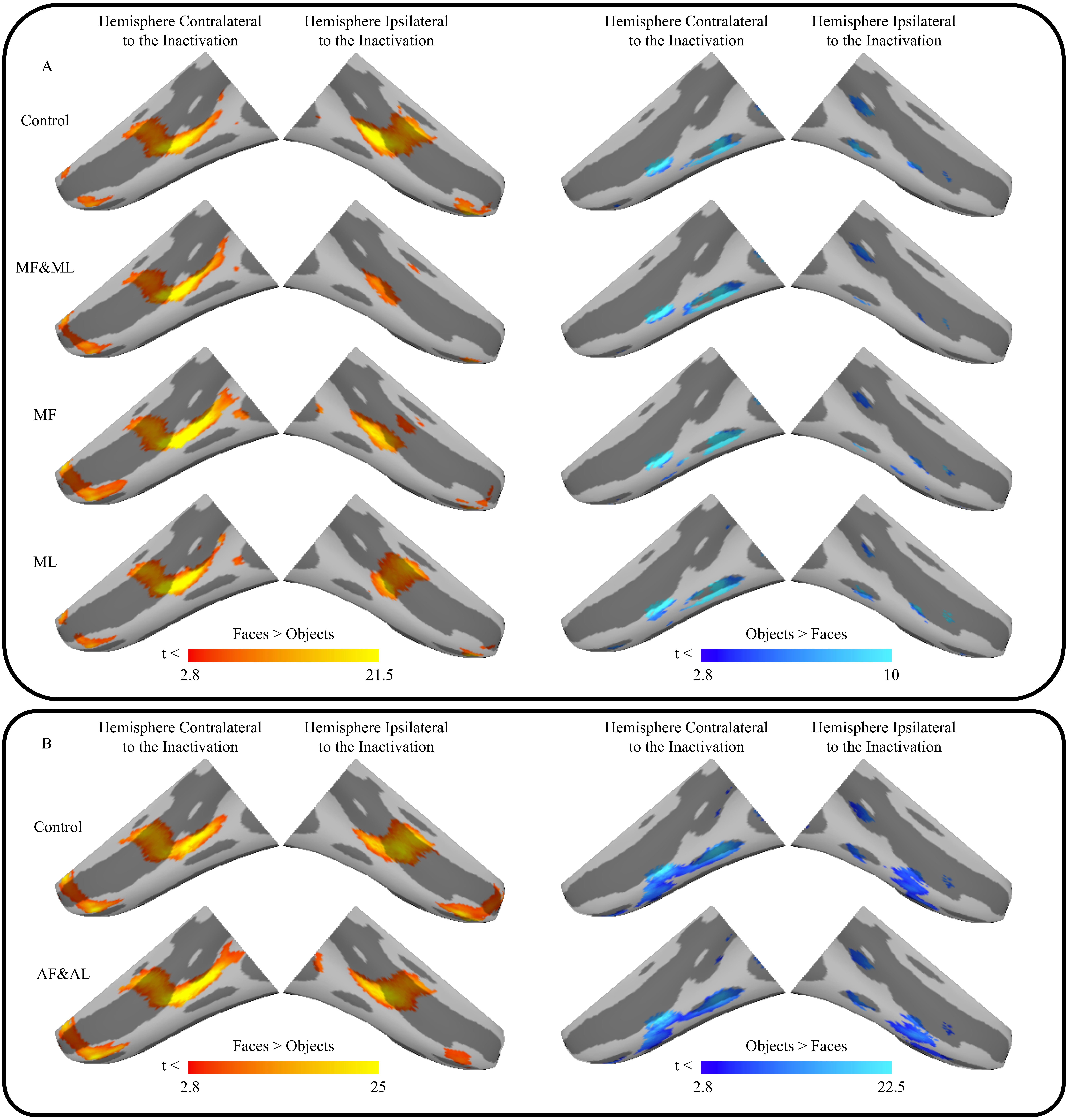

Figure S11. Examples of right middle face patches inactivation (A) and right anterior face patches inactivation (B) in Monkey C. Face-selective (left panels) and object-selective (right panels) activation maps (neutral monkey faces versus familiar objects, $p<0.005$ uncorrected) are shown on lateral views of both hemispheres of the inflated cortex. MF&ML: combined MF (middle fundal patch) and ML (middle lateral patch) inactivation; MF: MF alone inactivation; ML: ML alone inactivation; AF&AL: combined AF (anterior fundal patch) and AL (anterior lateral patch) inactivation.

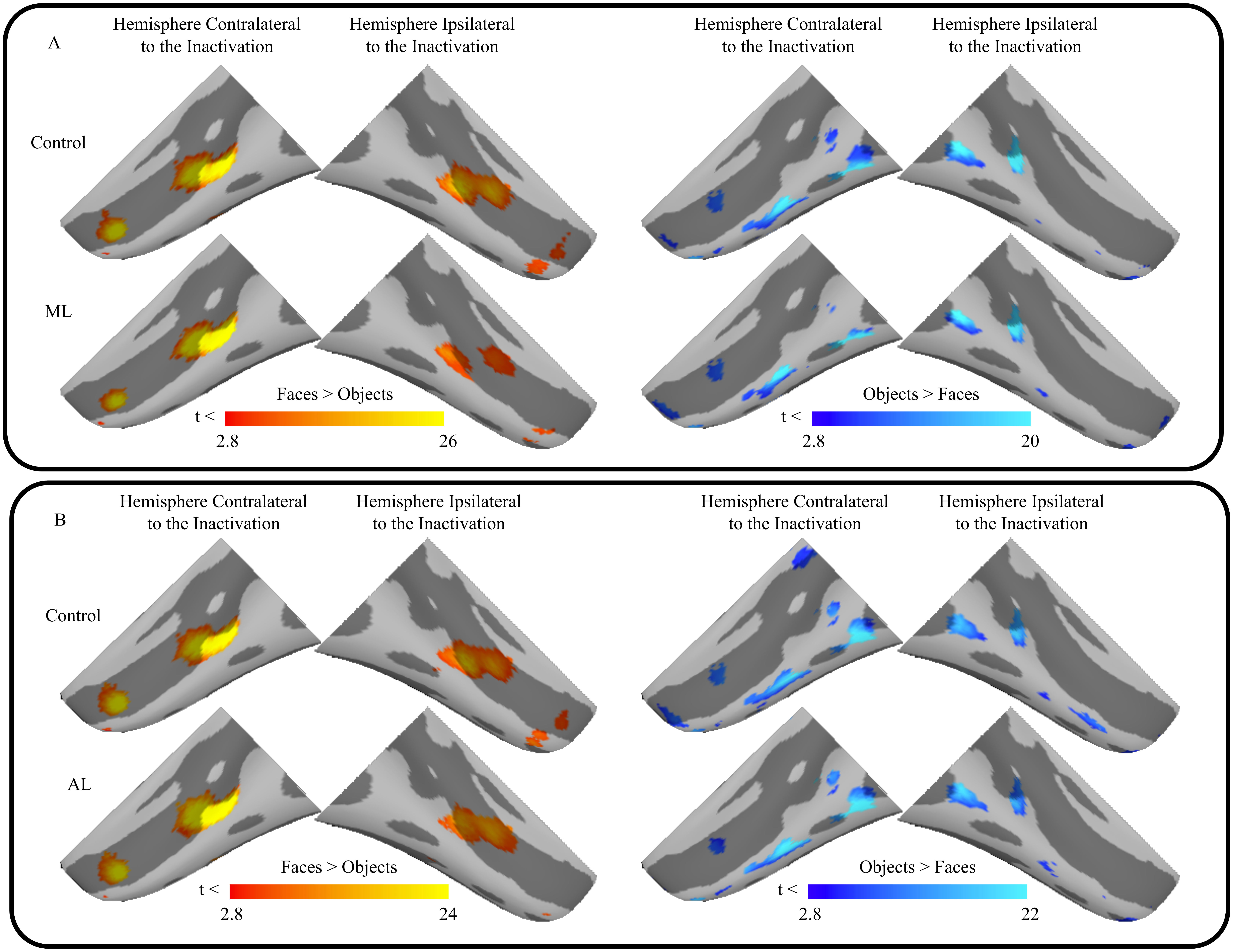

Figure S12. Examples of right middle face patch (ML) inactivation (A) and right anterior face patch (AL) inactivation (B) in Monkey D. Face-selective (left panels) and object-selective (right panels) activation maps (neutral monkey faces versus familiar objects, $p<0.005$ uncorrected) are shown on lateral views of both hemispheres of the inflated cortex. ML: ML (middle lateral patch) alone inactivation; AL: AL (anterior lateral patch) alone inactivation.

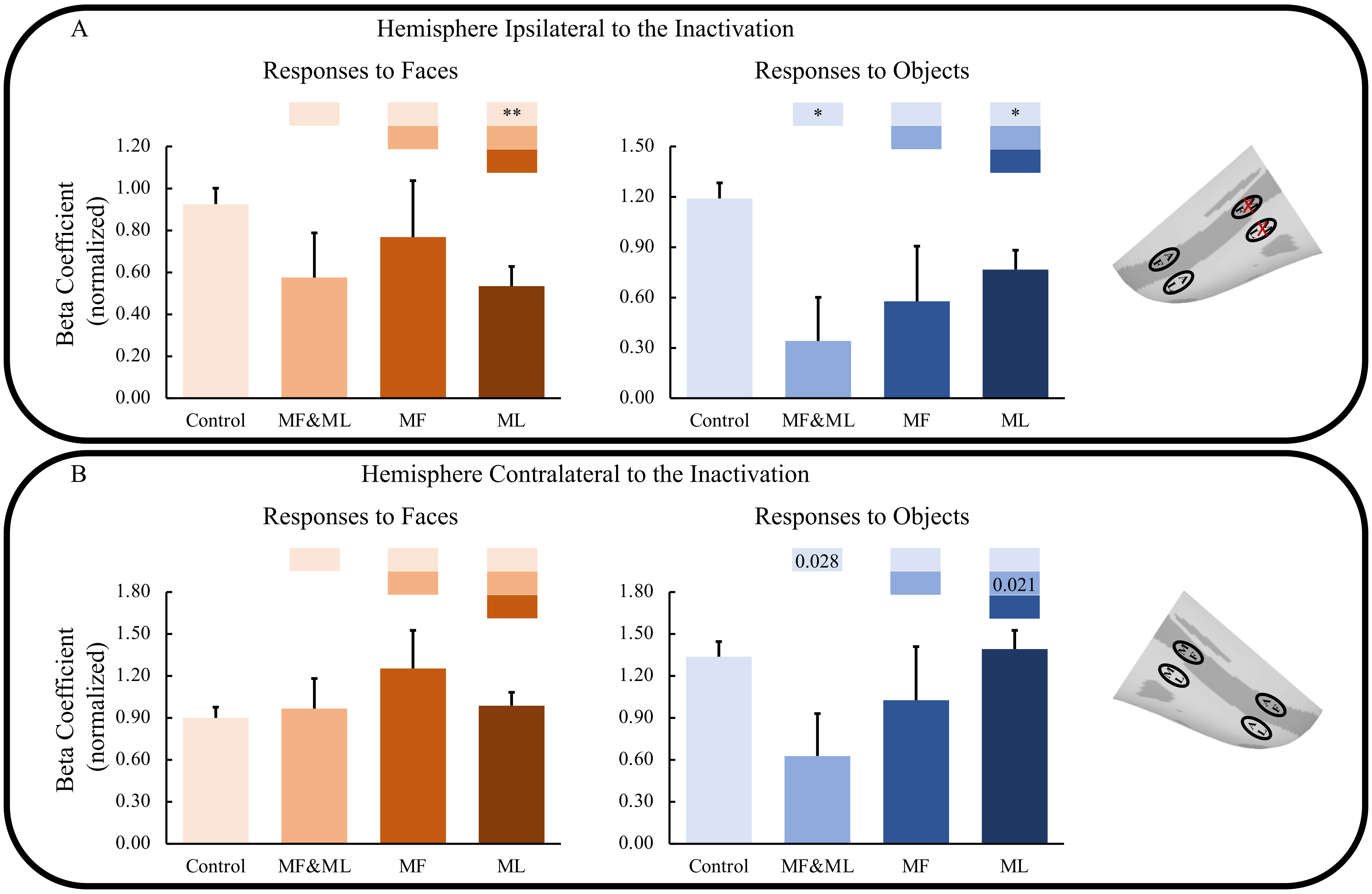

Figure S13. Effects of middle face patches inactivation on responses to faces and objects in the amygdala in the hemispheres ipsilateral (A) and contralateral (B) to the inactivation sites across both monkeys. MF&ML: combined MF and ML inactivation; MF: MF alone inactivation; ML: ML alone inactivation. Generalized Linear Mixed Models were performed with Treatment (Control: n=126, MF&ML: n=16, MF: n=10, ML: n=82) as fixed factors, and Session and Run as random factors. Post hoc testing was done with correction for multiple testing using the Holm-Bonferroni method. Color bars above a particular histogram contain the statistical results of comparing this inactivation condition and the condition to its left with the same color as the bar: *, significant difference from control (* p<0.05, ** p<0.01, in light red/blue boxes); value, uncorrected p value; empty bar, no significant difference no matter whether multiple comparison corrections were done or not. Box plots display mean values +/- SEM. The location of the inactivation is marked with red X. AF, anterior fundal patch; AL, anterior lateral patch; MF, middle fundal patch; ML, middle lateral patch.

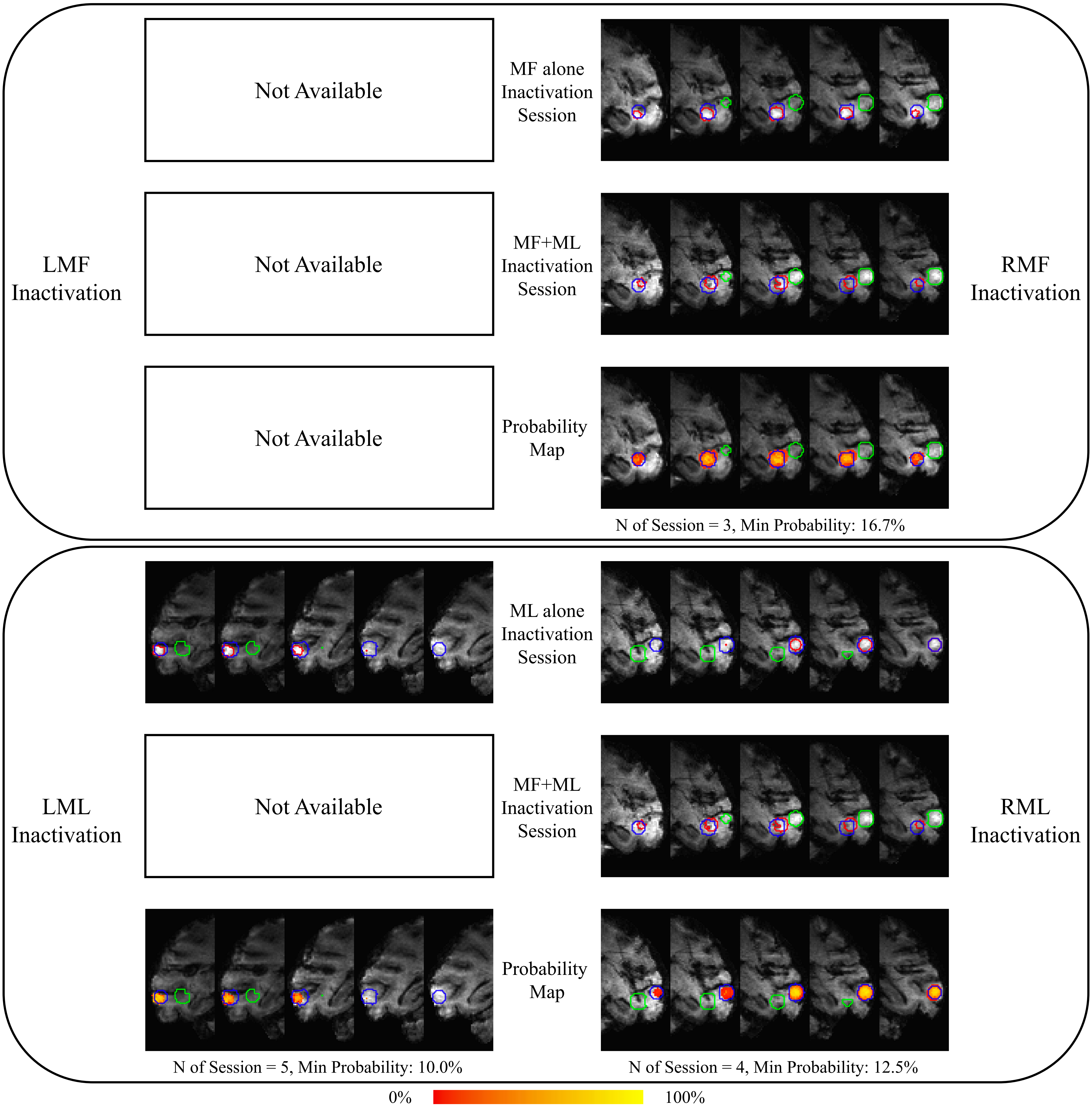

Figure S14. Examples and probability maps of Gd spread of middle face patches inactivation in Monkey C. The inactivation sites were evaluated by co-infused gadolinium, which could be visualized as a bright volume in post-injection anatomical scans. The borders of targeted ROIs are indicated by the blue lines, while the borders of nearby non-targeted ROIs are indicated by the green lines. The borders of Gd injection spread are indicated by the red lines. The probability maps of Gd spread were calculated by the average percentage intensity (divided by the maximum value of Gd brightness) across all the sessions. The minimum probability is 50% (the threshold for defining the Gd spread) divided by the number of sessions. AF, anterior fundal patch; AL, anterior lateral patch; MF, middle fundal patch; ML, middle lateral patch.

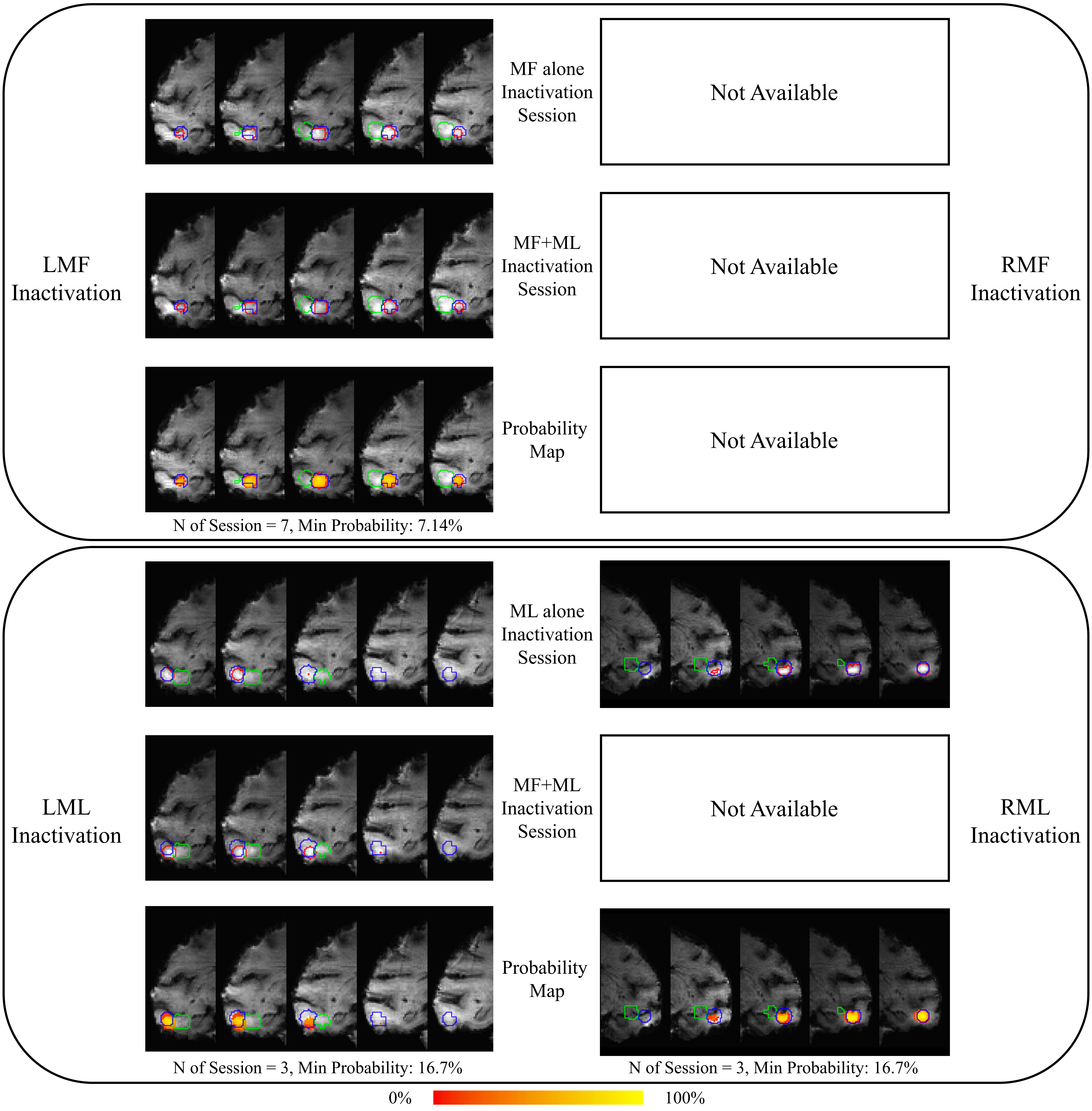

Figure S15. Examples and probability maps of Gd spread of middle face patches inactivation in Monkey D. The inactivation sites were evaluated by co-infused gadolinium, which could be visualized as a bright volume in post-injection anatomical scans. The borders of targeted ROIs are indicated by the blue lines, while the borders of nearby non-targeted ROIs are indicated by the green lines. The borders of Gd injection spread are indicated by the red lines. The probability maps of Gd spread were calculated by the average percentage intensity (divided by the maximum value of Gd brightness) across all the sessions. The minimum probability is 50% (the threshold for defining the Gd spread) divided by the number of sessions. AF, anterior fundal patch; AL, anterior lateral patch; MF, middle fundal patch; ML, middle lateral patch.

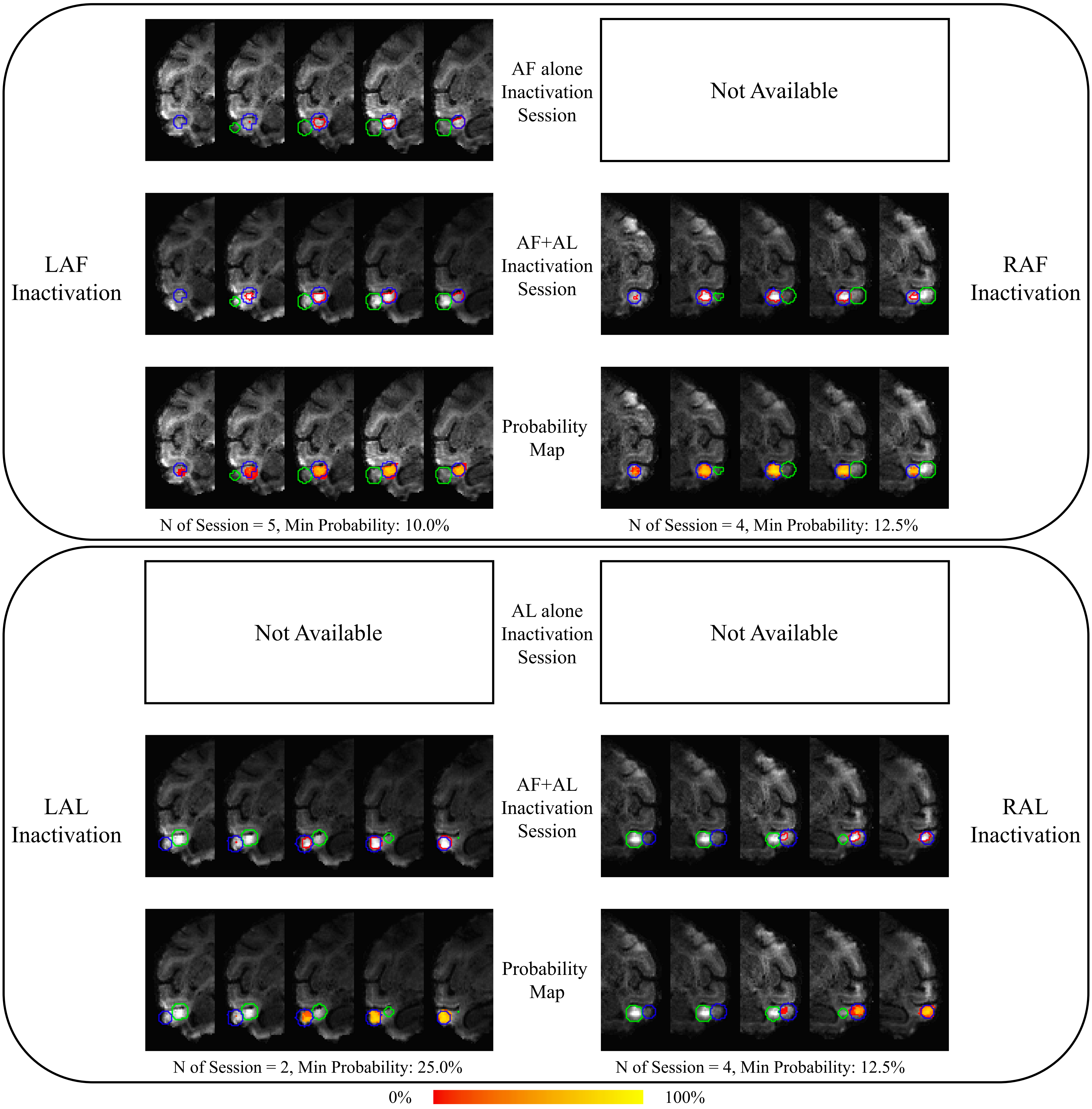

Figure S16. Examples and probability maps of Gd spread of anterior face patches inactivation in Monkey C. The inactivation sites were evaluated by co-infused gadolinium, which could be visualized as a bright volume in post-injection anatomical scans. The borders of targeted ROIs are indicated by the blue lines, while the borders of nearby non-targeted ROIs are indicated by the green lines. The borders of Gd injection spread are indicated by the red lines. The probability maps of Gd spread were calculated by the average percentage intensity (divided by the maximum value of Gd brightness) across all the sessions. The minimum probability is 50% (the threshold for defining the Gd spread) divided by the number of sessions. AF, anterior fundal patch; AL, anterior lateral patch; MF, middle fundal patch; ML, middle lateral patch.

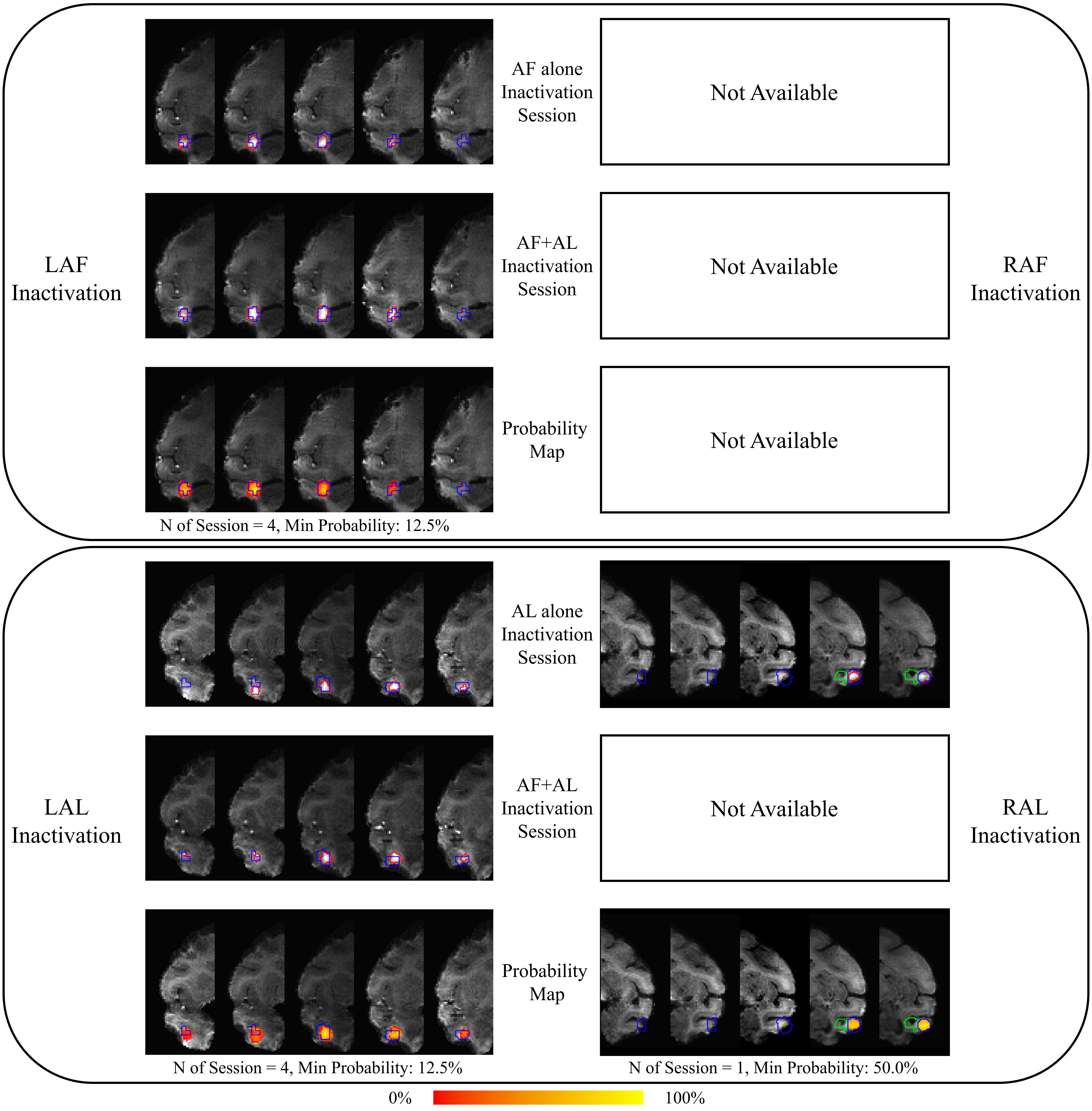

Figure S17. Examples and probability maps of Gd spread of anterior face patches inactivation in Monkey D. The inactivation sites were evaluated by co-infused gadolinium, which could be visualized as a bright volume in post-injection anatomical scans. The borders of targeted ROIs are indicated by the blue lines, while the borders of nearby non-targeted ROIs are indicated by the green lines. The borders of Gd injection spread are indicated by the red lines. The probability maps of Gd spread were calculated by the average percentage intensity (divided by the maximum value of Gd brightness) across all the sessions. The minimum probability is 50% (the threshold for defining the Gd spread) divided by the number of sessions. AF, anterior fundal patch; AL, anterior lateral patch; MF, middle fundal patch; ML, middle lateral patch.

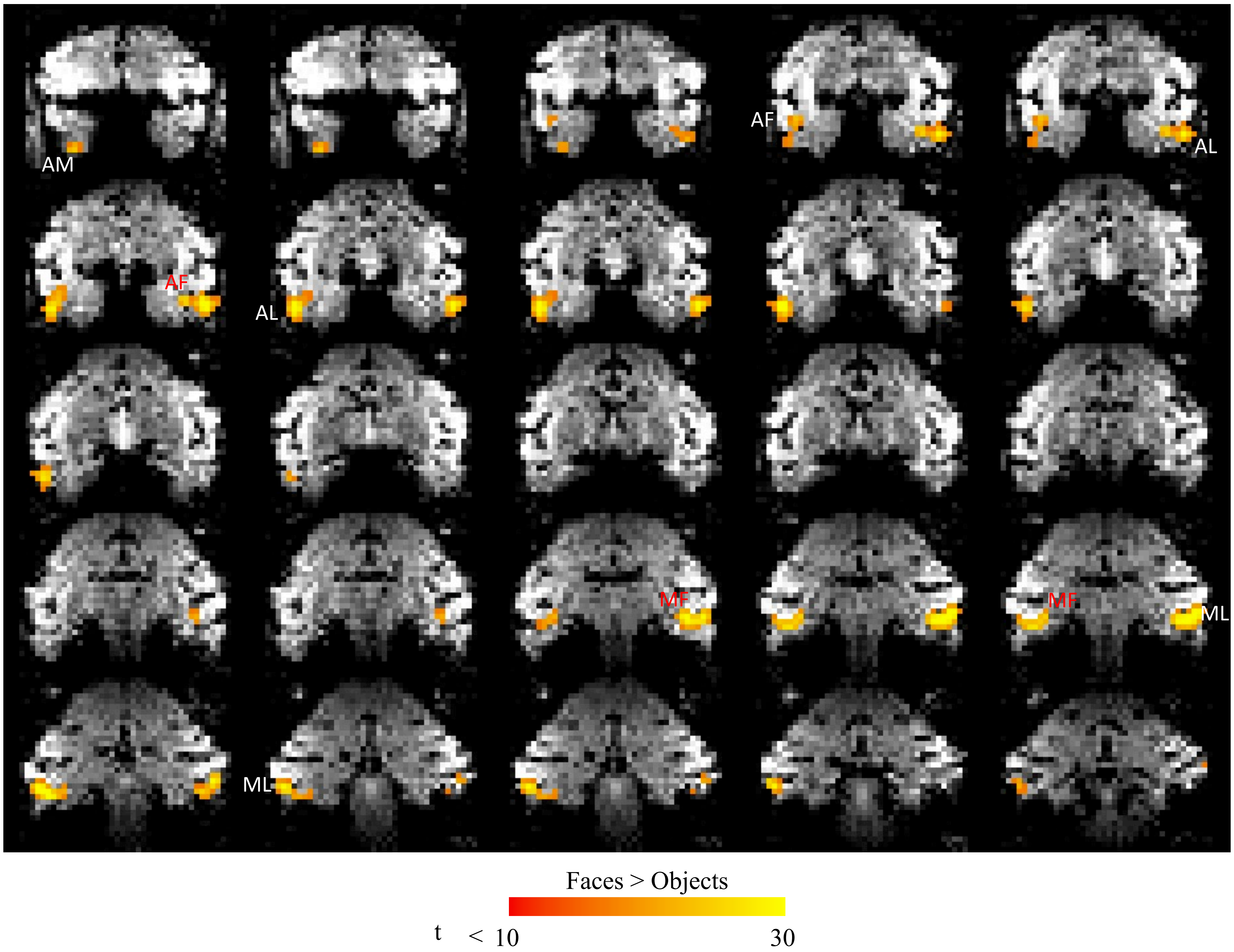

Figure S18. Face-selective patches in the left and right temporal lobes of Monkey C. Activation is overlaid on raw echo planar image (EPI slice). AF, anterior fundal patch; AL, anterior lateral patch; MF, middle fundal patch; ML, middle lateral patch.

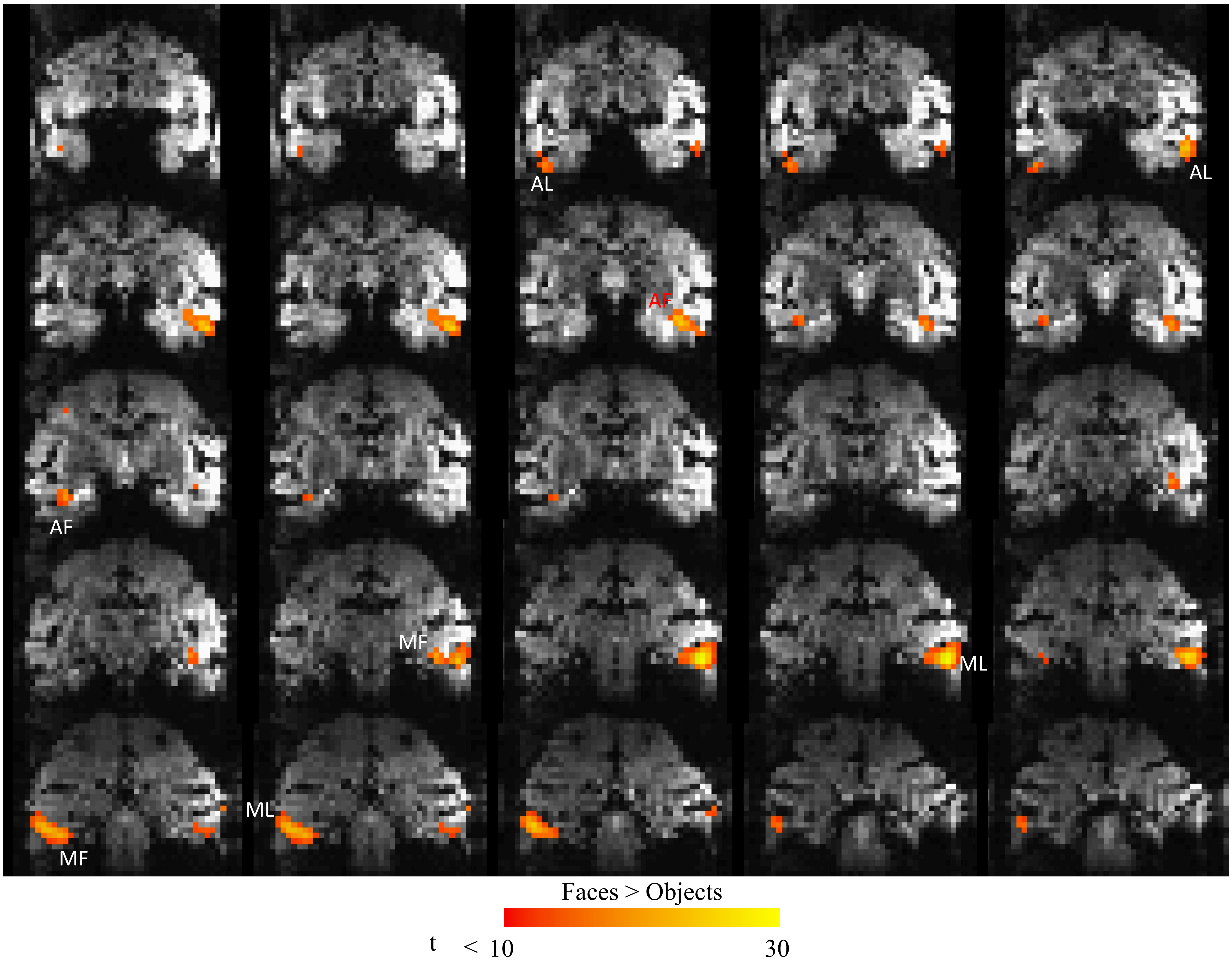

Figure S19. Face-selective patches in the left and right temporal lobes of Monkey D. Activation is overlaid on raw echo planar image (EPI slice). AF, anterior fundal patch; AL, anterior lateral patch; MF, middle fundal patch; ML, middle lateral patch.
