# Supplementary material for: Bidirectional and parallel relationships in macaque face circuit revealed by fMRI and causal pharmacological inactivation
Source: Nat Commun. 2022 Nov 9;13:6787. doi: 10.1038/s41467-022-34451-x (PMC9646786; doi:10.1038/s41467-022-34451-x)
Supplement: Supplementary file 3 — Reporting Summary [file 41467_2022_34451_MOESM3_ESM.pdf]

nature portfolio

Corresponding author(s): Ning Liu

Last updated by author(s): Oct 12, 2022

Reporting Summary

Nature Portfolio wishes to improve the reproducibility of the work that we publish. This form provides structure for consistency and transparency in reporting. For further information on Nature Portfolio policies, see our Editorial Policies and the Editorial Policy Checklist.

Statistics

For all statistical analyses, confirm that the following items are present in the figure legend, table legend, main text, or Methods section.

n/a	Confirmed	
☐	☒	The exact sample size (*n*) for each experimental group/condition, given as a discrete number and unit of measurement
☐	☒	A statement on whether measurements were taken from distinct samples or whether the same sample was measured repeatedly
☐	☒	The statistical test(s) used AND whether they are one- or two-sided<br>*Only common tests should be described solely by name; describe more complex techniques in the Methods section.*
☐	☒	A description of all covariates tested
☐	☒	A description of any assumptions or corrections, such as tests of normality and adjustment for multiple comparisons
☐	☒	A full description of the statistical parameters including central tendency (e.g. means) or other basic estimates (e.g. regression coefficient) AND variation (e.g. standard deviation) or associated estimates of uncertainty (e.g. confidence intervals)
☐	☒	For null hypothesis testing, the test statistic (e.g. *F*, *t*, *r*) with confidence intervals, effect sizes, degrees of freedom and *P* value noted<br>*Give P values as exact values whenever suitable.*
☒	☐	For Bayesian analysis, information on the choice of priors and Markov chain Monte Carlo settings
☐	☒	For hierarchical and complex designs, identification of the appropriate level for tests and full reporting of outcomes
☐	☒	Estimates of effect sizes (e.g. Cohen's *d*, Pearson's *r*), indicating how they were calculated

Our web collection on statistics for biologists contains articles on many of the points above.

Software and code

Policy information about availability of computer code

Data collection	QNX, Presentation (version 12.2)
Data analysis	MATLAB (R2016a), IBM SPSS (v24), AFNI (20.2.10)

For manuscripts utilizing custom algorithms or software that are central to the research but not yet described in published literature, software must be made available to editors and reviewers. We strongly encourage code deposition in a community repository (e.g. GitHub). See the Nature Portfolio guidelines for submitting code & software for further information.

Data

Policy information about availability of data

All manuscripts must include a data availability statement. This statement should provide the following information, where applicable:

- Accession codes, unique identifiers, or web links for publicly available datasets
- A description of any restrictions on data availability
- For clinical datasets or third party data, please ensure that the statement adheres to our policy

The data that support the findings of this study are available from the corresponding author, upon reasonable request.

Field-specific reporting

Please select the one below that is the best fit for your research. If you are not sure, read the appropriate sections before making your selection.

☒ Life sciences ☐ Behavioural & social sciences ☐ Ecological, evolutionary & environmental sciences

For a reference copy of the document with all sections, see nature.com/documents/nr-reporting-summary-flat.pdf

Life sciences study design

All studies must disclose on these points even when the disclosure is negative.

Sample size	The chosen sample size was referred to previous studies (Miyamoto et al., Science 2017; Sadagopan et al., eLife 2017; Bogadhi et al., Current Biology 2019).
Data exclusions	Data from those runs in which fixation was maintained on less than 90% of the runs were excluded.
Replication	Experimental procedures were performed in all four hemispheres from two animals. Results from individual animals and hemispheres showed similar patterns. No significant differences were found across monkeys or across hemispheres.
Randomization	Different pseudorandom sequences of categorical blocks were used in each run. The order of inactivation sites was randomized across monkeys and hemispheres.
Blinding	Blinding was not possible in the present study. Since muscimol-induced inactivations were conducted in the present study, for animal safety, we needed to what had been injected into the brain.

Reporting for specific materials, systems and methods

We require information from authors about some types of materials, experimental systems and methods used in many studies. Here, indicate whether each material, system or method listed is relevant to your study. If you are not sure if a list item applies to your research, read the appropriate section before selecting a response.

Materials & experimental systems

n/a	Involved in the study	
☒	☐	Antibodies
☒	☐	Eukaryotic cell lines
☒	☐	Palaeontology and archaeology
☐	☒	Animals and other organisms
☒	☐	Human research participants
☒	☐	Clinical data
☒	☐	Dual use research of concern

Methods

n/a	Involved in the study	
☒	☐	ChIP-seq
☒	☐	Flow cytometry
☐	☒	MRI-based neuroimaging

Animals and other organisms

Policy information about studies involving animals; ARRIVE guidelines recommended for reporting animal research

Laboratory animals	Macaca mulatta, Male, 9 years old
Wild animals	The study did not involve wild animals.
Field-collected samples	The study did not involve samples collected from the field.
Ethics oversight	National Institute of Mental Health Animal Care and Use Committee

Note that full information on the approval of the study protocol must also be provided in the manuscript.

Magnetic resonance imaging

Experimental design

Design type	Block design
Design specifications	To identify Regions of Interest (ROIs), we performed an initial localizer experiment as described previously29,41. Briefly, for Monkey C, grayscale photos of neutral monkey faces, familiar places, familiar objects, Fourier-phase scrambled

faces, Fourier-phase scrambled places, and Fourier-phase scrambled objects were presented in separate blocks (see Fig. 1A for example stimuli). Each block lasted 30 s and was presented once in each run. For Monkey D, grayscale photos of neutral monkey faces, familiar places, familiar objects, and Fourier-phase scrambled faces were presented in separate blocks. Each block lasted 32 s and was presented twice in each run.
In the subsequent inactivation and corresponding control experiments, to optimize the statistical power, only Grayscale photos of neutral monkey faces and familiar objects were presented to the animals. Each categorical block lasted 32 s and was presented four times in each run. In all experiments, each categorical block alternated with 20-s fixation blocks. Individual runs began and ended with a fixation block. Different pseudorandom sequences were used in each run. In each categorical block, 16 images were each presented for 700 ms followed by a 300-ms interval and repeated twice.

Behavioral performance measures

The monkeys were required to maintain fixation on the red square superimposed on the stimuli to receive a liquid reward. Data from only those runs in which fixation was maintained on at least 90% of the runs were included.

Acquisition

Imaging type(s)

functional and structural MRI

Field strength

4.7 T

Sequence & imaging parameters

Twenty-eight 1.5-mm coronal slices (no gap) were acquired using single-shot interleaved gradient-recalled echo planar imaging. Imaging parameters were as follows: voxel size: 1.5 mm isotropic, field of view: 96 × 54 mm; matrix size: 64 × 36; echo time (TE): 13.8 ms; repetition time (TR): 2 s; flip angle: 90°. A low-resolution anatomical scan was also acquired in each session to serve as an anatomical reference (modified driven equilibrium Fourier transform sequence, voxel size: 1.5 × 0. 5 × 0.5 mm; field of view: 96 × 96 mm; matrix size: 192 × 192; TE: 3.95 ms; TR: 11.25 ms; flip angle: 12°). To facilitate cortical surface alignment and the following local targeting of regions, we also acquired high-resolution T1-weighted whole-brain anatomical scans in separate sessions, using the modified driven equilibrium Fourier transform sequence. Imaging parameters were as follows: voxel size: 0.5 mm isotropic; TE: 4.1 ms; TR: 12 ms; flip angle: 12°.

Area of acquisition

For fMRI, twenty-eight 1.5-mm coronal slices (no gap) were acquired, which mainly covered V4, the entire temporal lobe and part of the prefrontal cortex.

Diffusion MRI ☐ Used ☒ Not used

Preprocessing

Preprocessing software

Functional data were preprocessed using Analysis of Functional NeuroImages software (AFNI, 20.2.10). Images were realigned to the base volume of one initial localizer session. Then, the data were smoothed with a 2-mm full-width half-maximum Gaussian kernel. Signal intensity was normalized to the mean signal value within each run.

Normalization

Images were realigned to the base volume of one initial localizer session. Linear transformation were conducted.

Normalization template

n/a

Noise and artifact removal

For each voxel, we performed a single univariate linear model fit to estimate the response amplitude for each condition. The model included a hemodynamic response predictor for each category and regressors of no interest (baseline, movement parameters, and signal drifts).

Volume censoring

Volumes where more than 0.5% of the brain voxels were computed as outliers were censored. No volume was censored out in the remained runs.

Statistical modeling & inference

Model type and settings

For each voxel, we performed a single univariate linear model fit to estimate the response amplitude for each condition.

Effect(s) tested

To explore the differences in treatment and hemisphere, we performed Generalized Linear Mixed Models (GLMMs) for the data from inactivations of middle face patches and anterior face patches separately. We treated Treatment (Placebo, F inactivation, L inactivation, and combined F and L inactivation) and Hemisphere (ipsilateral and contralateral hemisphere) as fixed factors, while treating monkey (C and D), L-R hemisphere (Left and right), session, and run as random factors, followed up with post hoc tests on responses to faces/object in each ROI. Post-hoc comparisons were adjusted for multiple testing using the Holm-Bonferroni method.

Specify type of analysis: ☐ Whole brain ☐ ROI-based ☒ Both

Anatomical location(s)

Amygdala, two middle face patches near area TEO, one located in the fundus of the superior temporal sulcus (STS) ("MF," for middle fundus) and one on the lower lip of the STS ("ML," for middle lateral); and two patches anteriorly in area TE, one located near the fundus of the STS ("AF," for anterior fundus), and one on the lower lip of the STS ("AL," for anterior lateral).

Statistic type for inference
(See Eklund et al. 2016)

The voxel-wise false discovery rate (FDR) approach (<0.05) was used.

Correction

Post-hoc comparisons were adjusted for multiple testing using the Holm-Bonferroni method.

Models & analysis

n/a	Involved in the study
☒	☐ Functional and/or effective connectivity
☒	☐ Graph analysis
☐	☒ Multivariate modeling or predictive analysis

Multivariate modeling and predictive analysis

We conducted linear regression analyses with responses to faces/objects across all the runs/sessions/treatments/monkeys in one ROI as the outcome and responses to faces/objects in the other ROIs as predictors.
